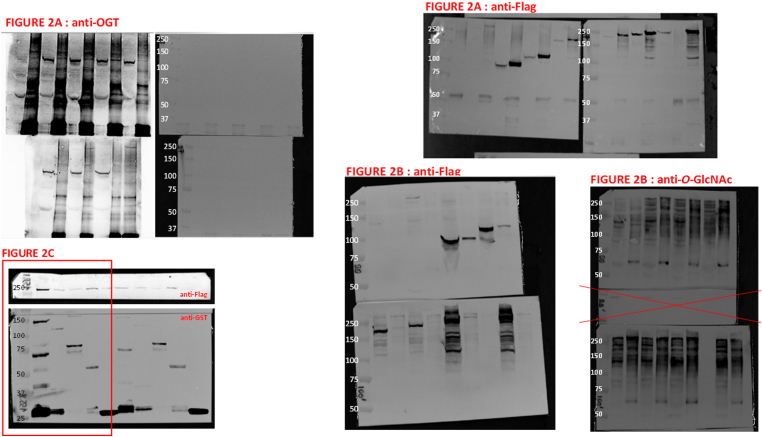# Mammalian fatty acid synthase and *O*-GlcNAc transferase preferentially interact *via* their respective *N*-terminal regions

**DOI:** 10.1016/j.bbrep.2025.102427

**Published:** 2026-01-06

**Authors:** Dimitri Vanauberg, Céline Schulz, Guillaume Brysbaert, Nessim Raouraoua, Peggy Mistarz-Gruau, Marc F. Lensink, Anne-Sophie Vercoutter-Edouart, Tony Lefebvre

**Affiliations:** University of Lille, CNRS, UMR 8576 - UGSF - Unité de Glycobiologie Structurale et Fonctionnelle, Lille, F-59000, France

**Keywords:** Fatty acid synthase, *O*-GlcNAc transferase, *O*-GlcNAcylation, *N*-terminal region, Liver cancer cells

## Abstract

Fatty Acid Synthase (FASN) is a central enzyme in the *de novo* lipogenesis pathway. By producing fatty acids, FASN is implicated in numerous crucial cellular processes, but it is also frequently overexpressed in cancer. *O*-GlcNAc Transferase (OGT) governs the addition of N-acetylglucosamine residues onto cytosolic, nuclear and mitochondrial proteins. Like FASN, OGT actively participates in carcinogenesis. We previously showed that OGT regulates FASN in different *ex vivo* and *in vivo* models. Reciprocally, FASN promotes OGT expression and activity. The two enzymes physically interact together and contribute to cancer cell survival. It is therefore fundamental to define the respective interaction region of each enzyme to explore new therapeutic solutions for patients suffering from cancer. By using the hepatocarcinoma cell line Hep3B, we show thanks to two series of deletion mutants that both enzymes preferentially interact *via* their respective N-terminal regions. Analysis of the *O*-GlcNAc status of the various FASN deletion mutants shows that stronger interaction with OGT correlates with higher glycosylation, suggesting that OGT catalyzes the transfer of GlcNAc with limited substrate specificity.

## Highlights

• FASN and OGT physically interact together *via* their respective N-terminal region.
• More OGT interacts with a N-terminus deletant version of FASN, more this construct is glycosylated.
• Modeling with AlphaFold reveals the interaction surface of both partners.

## 1 Introduction

**Fig. 1:**
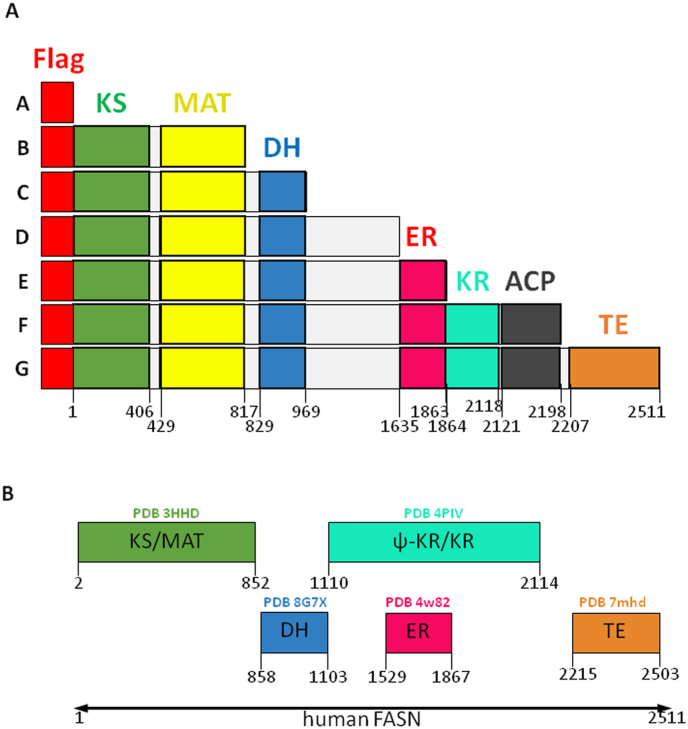
FASN constructions used in this study. **A.** The delimitation of each domain is indicated by numbering the amino-acids. Each construction encodes a Flag-peptide located at the N-terminus. The domain boundaries shown are based on the UniProt primary sequence annotation (fatty acid synthase access number P49327). **B.** Domain boundaries shown are based on available experimental structures (PDB IDs: 3HHD, 8G7X, 4PIV, 4w82 and 7mhd) ACP, Acyl Carrier Protein; DH, DeHydrogenase; ER, Enoyl-Reductase; KR, Keto-acyl Reductase; KS, Keto-acyl Synthase; MAT, Malonyl/Acetyl Transferase; TE, ThioEsterase.

FASN is a large enzyme, widely expressed in living organisms and active as a homodimer in animals [1,2]. Although a major fraction of the enzyme resides in the cytosol, a small fraction interacts with lipid rafts [3]. FASN requires no less than 7 catalytic activities to produce fatty acids: keto-acyl synthase (KS), malonyl/acetyl transferase (MAT), dehydrogenase (DH), enoyl-reductase (ER), keto-acyl reductase (KR) and thioesterase (TE) (Fig. 1). The ACP (acyl carrier protein) domain is necessary for substrate shuttling between the different catalytic domains through its phosphopantetheine group, covalently attached to S2156. After esterification, fatty acids constitute a major energy reserve in the form of triglycerides. Fatty acids are also key structural elements of biological membranes, act as acylation moieties for proteins, and play a central role in the production of several second messengers [4]. This explains why FASN is highly active in cell proliferation and pivotal in the processes of carcinogenesis [5]. The FASN expression level is increased by *O*-β-N-acetyl-D-glucosaminylation (*O*-GlcNAcylation) [6], a reversible post-translational modification (PTM), the dynamics of which is regulated by a unique pair of enzymes, *O*-GlcNAc transferase (OGT) and *O*-GlcNAcase (OGA) [7]. OGT adds N-acetyl-d-glucosamine (GlcNAc) groups from the nucleotide-sugar UDP-GlcNAc onto serine and threonine moieties of literally thousands of cytosolic, nuclear and mitochondrial proteins, while OGA hydrolyses the residues off the proteins in order to remove the PTM [8,9]. Human OGT contains 13.5 tetratricopeptide repeats (TPRs) in its N-terminal domain [10]. TPRs are degenerated consensus sequences of 34 amino acids found in many proteins that serve as anchors for protein-to-protein interaction. At the C-terminal end, OGT displays two distinct catalytic domains (CD), CDI and CDII, in addition to a nuclear localization sequence (NLS) [11]. As is the case with FASN, OGT is highly expressed in a large variety of cancers where it contributes to the processes of carcinogenesis [12].

Because of their pro-oncogenic activities, defining the respective domains of FASN and OGT that drive their interaction is of special interest so as to be able to propose novel therapeutic avenues for cancer treatment, especially hepatocarcinomas. In previous studies, we showed that FASN and OGT physically interact both *in vivo* and *ex vivo* [6]. *O*-GlcNAcylation of FASN decreases its ubiquitination by promoting FASN interaction with the deubiquitinase Ubiquitin Specific Peptidase 2a (USP2a) which reduces its sensitivity to the proteasome. Moreover, inhibition of FASN leads to a decrease in OGT level and, in turn, inhibition of OGT reduces the level of FASN, with the consequence of a deregulated cell cycle and a decreased cell viability [13]. To promote cancer cell survival, FASN has been shown to inhibit OGA under conditions of oxidative stress [14], whereas *O*-GlcNAcylation enhances FASN activity during nutrient starvation [15]. Li and collaborators [16] highlighted interaction of FASN with OGT in mouse hepatocytes (AML-12 cells); these authors showed that *O-*GlcNAcylation at S1483 – located between the DH and KR regions - reduces FASN K48-ubiquitination and degradation, increasing its activity and subsequent hepatic lipid accumulation. Altogether, these data contribute to shedding light onto the complex relationship between FASN and OGT.

In the present study, by combining co-immunoprecipitations, GST pull-down, and structural modeling, we revealed a preferentially *N*-terminal-to-N-terminal interaction between FASN and OGT. These findings may lead to future identification of FASN-OGT interaction inhibitors as potential anti-cancer therapeutics.

## 2 Materials and methods

### 2.1 Cell culture and transfection

The hepatocarcinoma Hep3B human cell line came from the ONCOLille Institute (Lille) and is certified by the ATCC. Cells were cultured in Minimal Essential Medium (MEM, Biowest) supplemented with 2 mM l-glutamine, 1 mM sodium pyruvate, 10 % (v/v) fetal calf serum and penicillin/streptomycin at 1:100 dilution (Gibco) and placed at 37 °C, in a 5 % (v/v) CO_2_-enriched humidified atmosphere. Cells were transiently transfected for 24 h with 10 μg of plasmids in 150 cm^2^ flasks using jetOPTIMUS (Polyplus).

#### 2.1.1 Cell lysis, SDS-PAGE and Western blotting

After washing in ice-cold phosphate-buffered saline (PBS), Hep3B cells were lysed in lysis buffer (50 mM Tris/HCl, 150 mM NaCl, 0.5 % NP-40 (v/v), 50 mM sodium fluoride, 1 mM sodium orthovanadate, protease inhibitors, pH 8.0) for 10 min. The supernatants were collected by centrifugation at 20,000×*g* for 15 min at 4 °C. Protein content was evaluated using the micro-BCA protein assay kit (Thermo Fisher Scientific). Samples were mixed with Laemmli buffer (250 mM Tris-HCl, 5 % (w/v) SDS, 5 % (v/v) 2-mercaptoethanol, 40 % (v/v) glycerol, pH 6,8), and heated to 95 °C for 7 min. Proteins were separated by 8 % SDS-PAGE in electrophoresis buffer (25 mM Tris–HCl, 192 mM glycine, 0.1 % (w/v) SDS, pH 8.8), and electro-transferred onto nitrocellulose (HybondTM-C EXTRA, GE Healthcare) in transfer buffer (25 mM Tris–HCl, 192 mM glycine, 20 % (v/v) methanol, pH 8.8). After staining with Ponceau red (5 % (v/v) acetic acid and 0.1 % (w/v) Ponceau red) to check efficiency of the transfer and equal loading, membranes were destained in TBS (Tris-Buffered Saline)-Tween20 (20 mM Tris–HCl, 150 mM NaCl, 0.05 % (v/v) Tween20 (Sigma-Aldrich), pH 7.5) (TBS-T), blocked in 5 % (w/v) nonfat dry milk in TBS-T, and incubated overnight at 4 °C with primary antibodies (Supp. Table 1). Membranes were washed in TBS-T three times and then incubated with either anti-mouse or anti-rabbit horseradish peroxidase-conjugated secondary antibody (Supp. Table 1) for 1 h at room temperature (RT). Membranes were washed again in TBS-T three times and signal was detected using enhanced chemiluminescence (West Pico Plus or Femto, ThermoScientifc). Images were captured using a CCD camera (Fusion Solo, Vilbert Lourmat) and analyzed using Image J software to measure optical densities.

### 2.2 Co-immunoprecipitation

Hep3B cells were incubated in lysis buffer as described above. Lysates containing 1 mg of total protein were first pre-cleared by incubation with 100 μL of a 50/50 mix of A/G protein-coupled agarose beads (Cytiva) for 1 h at 4 °C. The supernatants were collected following gentle centrifugation of the beads, and incubated with 2.5 μg of an anti-Flag antibody (Merck, mouse monoclonal anti-Flag M2, F1804) or the same amount of mouse IgG-UNLB (Southern Biotech) as a control. Thirteen and 40 μL agarose-beads coupled to A or G proteins respectively were incubated with the protein samples for 1 h at 4 °C. Beads were washed three times with lysis buffer and heated to 95 °C in Laemmli buffer for 7 min.

### 2.3 WGA-beads enrichment

Hep3B cell lysates were diluted into lysis buffer to obtain a final dilution of 1 mg/mL of proteins and incubated with 20 μL of WGA-labeled beads (Vectolab) overnight at 4 °C (specificity of interaction was checked by adding 0.5 M of free GlcNAc). WGA-bound proteins were collected by spin-centrifugation, and beads were washed in the following buffer: 10 mM Tris/HCl, 100 mM NaCl, 0.4 % (w/v) sodium deoxycholate, 0.3 % (w/v) SDS, and 0.2 % (v/v) NP-40, pH 7.5, four times, and heated to 95 °C for 7 min in Laemmli buffer.

### 2.4 GST pull-down assay

The bacterial expression vectors pGEX-2T for GST and GST-OGT fusion proteins were generously provided by Pr. Xiaoyong Yang from the Yale University School of Medicine, CT. To produce the GST-recombinant proteins, the *E. coli* BL21 DE-3 strain was transformed with the vectors and cultured in a LB medium supplemented with ampicillin at 50 μg/mL. When exponential growth phase was attained, protein production was induced by addition of 0.1 mM IPTG for 4 h at RT. Bacteria were harvested by centrifugation and re-suspended in PBS supplemented with protease inhibitors (Sigma-Aldrich). Bacteria were lysed by high-pressure homogenization (Emulsiflex-C3, Avestin, Mannheim, Germany). A centrifugation was performed at 2400×*g* for 10 min, and GST-fused proteins were bound onto Glutathione Sepharose 4B beads (GE Healthcare) for 2 h at 4 °C. Beads were washed for 5 min in a buffer containing 20 mM Tris at pH 7.5 first, then two times with 20 mM Tris pH 7.5 added to 0.1 % (v/v) Triton X-100 and 100 mM NaCl, twice, and finally with 50 mM Tris pH 8.0. Then, 700 μg of protein from Hep3B cells overexpressing 3xFlag-FASN were added with the beads and gentle incubation was done for 2 h at 4 °C. Bound proteins were washed first in PBS, then three times in PBS added to 0.1 % (v/v) Triton X-100, in PBS added to 0.1 % (v/v) Triton X-100 and 150 mM NaCl, and finally in 50 mM Tris at pH 8.0. The bound proteins were eluted by incubating beads with 50 μL of elution buffer (50 mM Tris (pH 8.0) with 50 mM reduced glutathione (Sigma-Aldrich) for 10 min by gentle vortex.

### 2.5 FASN cloning and generation of the deletion mutants

FASN gene was cloned into the pCMV10-3xFlag expression vector thanks to the *Hin*d III - FASN sense primer and FASN - *Xba*I antisense primer (Eurogentec) (Supp. Table 2). Domain boundaries of FASN were mainly defined based on the UniProt database annotation (UniProt: P49327). The construction of the various FASN deletion mutants was allowed by introducing STOP codons at the desired position to abort protein synthesis (QuikChange II XL Site-Directed Mutagenesis Kit, Agilent). The primers used are listed in Supp. Table 2, and the chemicals were 1 μL dNTPs, 3 μL Quick Solution, 2.5 Unit Pfu turbo ultra, completed to 50 μL distilled water. PCR conditions were the following: 60 s at 95 °C, 18 denaturation cycles at 95 °C for 50 s, hybridization step at 60 °C for 50 s, elongation step at 68 °C for 14 min, and 7 min at 68 °C. At the end of the PCR procedure, the non-mutated parent vector was digested by incubating the PCR mix with *Dpn*I enzyme for 1 h at 37 °C.

### 2.6 Bacteria transformation

Transformation of *E. coli* (XL10 Gold or DH5α strains) was carried out by incubating plasmids with bacteria (45 μL) in 2 μL of 2-mercaptoethanol for 10 min directly on ice. Two μL of the PCR product were added prior 30 min incubation at 4 °C, then 30 s at 42 °C, and finally 2 min at 4 °C. *E. coli* were placed in 500 μL of LB medium for 1 h at 37 °C under agitation before plating in Petri dishes containing LB agar supplemented with carbenicillin at a final concentration of 50 μg/mL.

### 2.7 Plasmids extraction and amplification

Extraction of plasmids was performed with the Plasmid DNA purification-nucleospin Plasmid kit from Macherey Nagel. Colonies were seeded in 5 mL of LB medium containing carbenicillin and maintained overnight at 37 °C. Bacteria were then centrifuged at 2,400×*g*. Plasmids were extracted following the recommendations of the manufacturer, and assay of DNA was done by measurement of optical density at 260 nm with a NanoDrop apparatus. After careful verification of the sequences (Eurofins Genomics), plasmids were amplified (NucleoBond Xtra-Midi kit, Macherey Nagel). At last, amounts of DNA were evaluated by NanoDrop assay.

### 2.8 FASN dimer and OGT structure prediction

The FASN dimeric structure (UniProt: P49327) was predicted using AlphaFold3 [17] with default parameters. ipTM and pTM scores are respectively 0.68 and 0.7 for the top ranked prediction. The OGT monomer (UniProt: O15294) was modeled before with AlphaFold2 with a mean pLDDT of 92.11. A complex made of a single FASN trimmed to residues 1–969 and a single OGT trimmed to residues 1–286 was modeled with AlphaFold3 to establish the interaction interface. 1000 predictions were computed with 200 seeds and 5 samples for each. The top ranked prediction was selected. ipTM and pTM scores are 0.58 and 0.78, respectively, for the top ranked prediction. The full OGT monomers and FASN dimer structures were then superimposed onto this complex. The rendering and superimpositions were done with PyMol 2.3.0 [18].

### 2.9 Statistical analysis

Data (means ± SEM) were analyzed by one-way ANOVA or Student's *t*-test (two-tailed and unpaired) using Graph-Pad Prism 8.0.2 (GraphPad Software, Inc.).

## 3 Results

### 3.1 FASN preferentially interacts with OGT *via* its N-terminal region

**Fig. 2:**
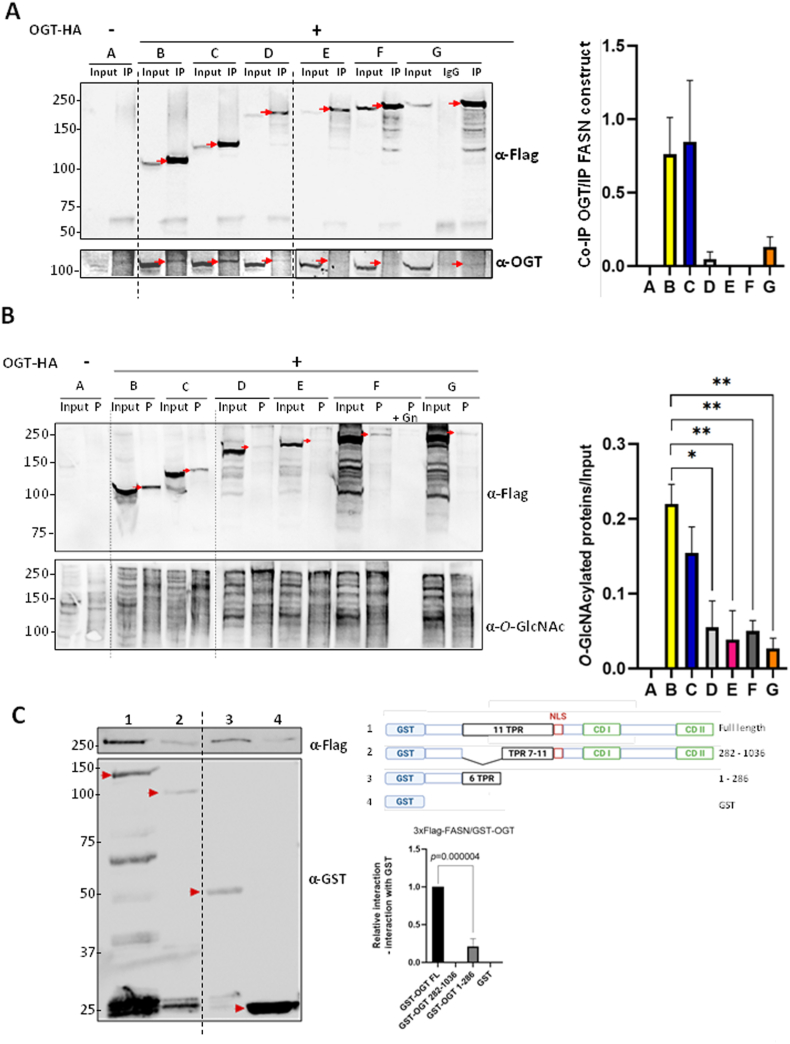
FASN and OGT interact *via* their respective N-terminal part. **A and B.** Hep3B cells were transfected with an empty vector (A) or with the different 3xFlag-FASN deletion mutants (B to G) in combination with OGT-HA overexpression. Protein samples were incubated with an anti-Flag antibody for co-immunoprecipitation with OGT (“IP” lanes for “Immuno-Precipitated”) (**panel A**) or with WGA-agarose beads (“P” lanes for “Purified”) (**panel B**). **A.** Co-immunoprecipitation of the deletion mutants with OGT analyzed by Western blotting. Optical densities of co-IP OGT were measured and normalized with the corresponding IP deletion mutants of FASN (red arrows; n = 3) (arbitrary units). **B.***O*-GlcNAcylation levels of the deletion mutants analyzed by Western blotting. Specificity of WGA was checked by adding 0.5 M GlcNAc (“P + Gn” lane). Optical densities of the “purified” *O*-GlcNAcylated constructs were measured and normalized with inputs (red arrows; n = 3) (arbitrary units). ∗*p* < 0.05; ∗∗*p* < 0.01. **C.** Hep3B cells were transfected with 3xFlag-FASN. Protein samples were incubated with a GST-OGT construct (1–3) or GST only (4). After GST pull-down, interaction of the GST-OGT constructs (red arrows) with 3xFlag-FASN was analyzed by Western blotting. Optical densities of pull-down 3xFlag-FASN were measured and normalized with the corresponding construct. Interaction with GST only was subtracted from the other bands (n = 4). CD, Catalytic Domain; GST, Gluthatione-S Transferase; NLS, Nuclear Localization Signal; TPR, TetratricoPeptide Repeats. Dotted lines indicate where the blots were cut and joined during figure preparation. Molecular mass markers are indicated on the left (kDa).

To determine which respective domains of FASN and OGT interact, we first engineered a series of 3xFlag-FASN deletion mutants (Fig. 1A, constructs B to G, A corresponds to the empty vector expressing only the FLAG peptide). Of note, it is recognized that structural data from the Protein Data Bank (PDB) indicates that actual structurally determined domain boundaries may differ by several residues from the UniProt primary sequence annotation: some of these PDB structures boundaries are shown in Fig. 1B. The constructs were transfected into Hep3B cells as described in the materials and methods section. Co-immunoprecipitations were performed with the anti-Flag antibody, and the interaction with OGT was evaluated by Western blot (Fig. 2A). Although OGT interacts with the full-length FASN (construct G), the stronger complexes were formed by the association of OGT with constructs B and C that correspond to the FASN N-terminal region comprising the KS, MAT and DH domains (according to our delimitation of the domains boundaries). In parallel with the co-IP experiments, we evaluated the ability of the various FASN deletion mutants to be *O*-GlcNAcylated. The glycosylation level of the different constructs was determined by Wheat Germ Agglutinin (WGA)-beads enrichment (Fig. 2B). Interestingly, the FASN deletion mutants that co-immuno-precipitate most strongly with WGA also correspond to constructs B and C. These observations demonstrate that increased interaction between OGT and a given FASN deletion mutant form correlates with higher glycosylation of that construct.

### 3.2 OGT preferentially interacts with FASN *via* its N-terminal region

Most of OGT substrates interact with its N-terminally located TPRs [10]. Thus, we conversely used GST-OGT deletion mutants [19] to determine where in the TPRs the interaction with FASN occurs. After production in *E. coli*, each construct (1–4) (Fig. 2C) was incubated with lysates prepared from Hep3B cells overexpressing 3xFlag-FASN, and then GST pull-down assays were performed (Fig. 2C). We detected a strong interaction between FASN and full-length OGT (lane 1) as previously reported by us and others [6,13,15,16]. Interestingly, OGT lacking the first six TPRs (construction 2) loses almost all its interaction with FASN (lane 2). Using only the first six TPRs (construction 3) showed an interaction with FASN (lane 3). Although the latter construction interacts less strongly with FASN than full-length OGT, these data highlight the requirement of OGT TPRs 1–6 for its interaction with FASN.

### 3.3 FASN and OGT N-terminal-to-N-terminal interaction modeling

**Fig. 3:**
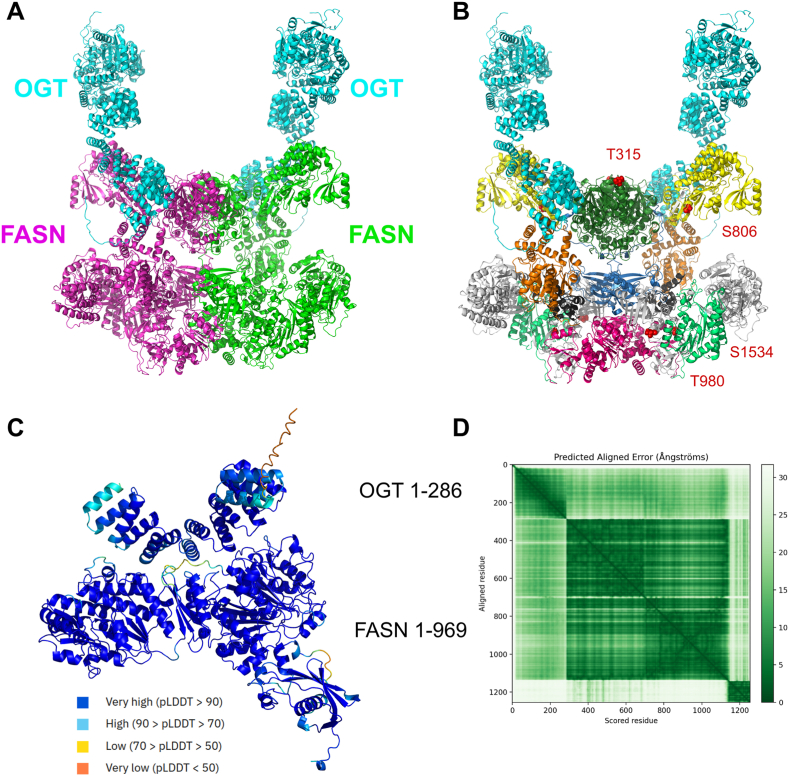
FASN and OGT interaction modeling. **A.** Modeling of FASN dimer and two OGT monomers; full sequences of the FASN dimer and of the OGT monomer were modeled independently with AlphaFold (see Materials and methods), assembled according to co-prediction performed in 3C and rendered in cartoon representation, FASN dimer in magenta and green (2511 residues each) and OGT monomers in cyan (1046 residues each). **B.** Same as **A,** but with FASN colored according to the domains depicted in Fig. 1 and the four *O*-GlcNAcylated sites previously identified and reported, T315, S806, T980 and S1534, highlighted by red spheres [8]. **C.** Model of FASN *N*-ter (bottom domain) and OGT *N*-ter (top domain, the TPR helices are clearly visible); the FASN sequence was trimmed to 1–969 and the OGT sequence to 1–286; the structure was predicted using AlphaFold3 and rendered in cartoon representation; residues are colored according to the pLDDT confidence score. **D**. Predicted Aligned Error matrix of the complex in **C**, ordered as OGT first and then FASN.

Inspired by our bench experiments, we used the AlphaFold 3 software to model the N-terminal-to-N-terminal interaction between FASN and OGT, with the purpose of visualizing the molecular interface underlying their binding (Fig. 3). We reconstructed a full FASN dimer binding to two OGT monomers (Fig. 3A and B), based on the prediction of the delimited FASN region 1–969 binding to the six first TPRs of OGT (1–286) (Fig. 3C). This prediction (Fig. 3C) has a medium ipTM score of 0.58, and also the PAE matrix (Fig. 3D) shows a medium-level uncertainty for the relative position between the chains. The reconstruction of the full OGT/FASN dimer complex highlights that this binding mode does not create clashes in the structure, making it a plausible model despite a medium confidence in the positioning of the OGT relatively to FASN as shown in the PAE matrix of the trimmed sequences (Fig. 3C and D). The list of the interface residues between each partner is given in Supp. Table 3.

## 4 Discussion

The lipogenic enzyme FASN is considered a master hepatic pro-oncogenic enzyme since its expression level can be decisive in the initiation of tumorigenesis [5,20]. *O*-GlcNAcylation, and the enzyme responsible for this modification, OGT, are both usually increased in tumors and promote many hallmarks of cancer including hepatocellular carcinoma (HCC) [21,22]. Therefore, investigating the FASN-OGT interaction in HCC is of special interest. In the present report, we show that the N-terminal part of FASN interacts strongly with the N-terminal part of OGT, which contains TPR domains pivotal in the recruitment of OGT's substrates [10]. Although little is known about FASN interactions with protein partners, our data regarding OGT is consistent with previous reports [[23], [24], [25], [26]]. To go further, the mutation of specific amino acids at the FASN-OGT interface could help in identifying those holding a key position for the interaction. Furthermore, the observation that the stronger OGT interacts with the various FASN deletion mutants, the more these mutants are glycosylated (Fig. 2B), supports the idea that the glycosyltransferase exhibits limited substrate specificity, as its discrimination mechanism remains poorly understood. It is therefore likely that a third regulatory partner is required, with each OGT client relying on its own specific regulatory subunit. It was shown recently that FASN can be *O*-GlcNAc-modified at S1483, located between the DH and ER domains, with the authors arguing that this residue could serve as an interaction site between FASN and OGT [16]. Given the large size difference between the two enzymes (Fig. 3A and B), we can assume that, depending on the position of the FASN *O*-GlcNAcylated site, OGT must interact near each individual site to perform the glycosylation process. Future experiments on the interaction between OGT and constructs corresponding only to central domains of FASN could help answer this question.

To date, no FASN inhibitor has been approved for cancer treatment, toxicity and metabolic side effects remain potential concerns. However, protein-protein interaction (PPI) inhibitors have gained particular attention in oncology. For instance, the approval of venetoclax, an inhibitor of BCL2-BAX interaction, to treat leukemia [27], provides tangible evidence of the therapeutic potential of targeting PPIs. Hence, targeting the OGT-FASN interaction using this kind of therapeutic approach is of particular interest. Several studies report that *O*-GlcNAcylation of FASN increases its stability and activity [6,13,15,16]. Thus, disrupting the interaction between the two pro-oncogenic enzymes FASN and OGT should reduce FASN *O*-GlcNAcylation and consequently decrease its subsequent activity and pro-tumoral effects. This hypothesis requires intensive further investigation.

Lastly, due to the tight relationship between *O*-GlcNAcylation and *de novo* lipogenesis, investigating OGT-FASN interaction in pathologies associated with nutritional disorders would be of great interest. As a whole, these results could open exploration of targeting OGT-FASN interaction, and may offer potential novel therapeutic solutions for cancer patients.

## Data statement

All data generated or analyzed during this study are included in this published article. Any additional information is available upon reasonable request to the corresponding author.

## CRediT authorship contribution statement

**Dimitri Vanauberg:** Conceptualization, Data curation, Investigation, Methodology, Writing – original draft. **Céline Schulz:** Investigation, Methodology, Writing – review & editing. **Guillaume Brysbaert:** Investigation, Methodology, Writing – review & editing. **Nessim Raouraoua:** Investigation, Methodology, Writing – review & editing. **Peggy Mistarz-Gruau:** Investigation, Methodology, Writing – review & editing. **Marc F. Lensink:** Investigation, Methodology, Writing – review & editing. **Anne-Sophie Vercoutter-Edouart:** Investigation, Methodology, Writing – review & editing. **Tony Lefebvre:** Conceptualization, Data curation, Funding acquisition, Project administration, Supervision, Writing – original draft.

## Declaration of competing interest

The authors declare that they have no known competing financial interests or personal relationships that could have appeared to influence the work reported in this paper.

## Figures and Tables

**figs1:**