# Supplementary material for: Mammalian fatty acid synthase and O-GlcNAc transferase preferentially interact via their respective N-terminal regions
Source: Biochem Biophys Rep. 2026 Jan 6;45:102427. doi: 10.1016/j.bbrep.2025.102427 (PMC12808501; doi:10.1016/j.bbrep.2025.102427)
Supplement: Multimedia component 1 [file mmc1.docx]

**Supplementary table 1: Antibodies used in this study.**

| **Antibodies** | **Dilution for WB** |
| --- | --- |
| **Primary antibodies (supplier, reference)** | |
| Mouse monoclonal anti-*O*-GlcNAc (Thermo Scientific, RL2) | 1 : 1,000 |
| Rabbit polyclonal anti-OGT (Sigma-Aldrich, DM-17) | 1 : 1,000 |
| Mouse monoclonal anti-Flag M2 (Sigma-Aldrich, F1804) | 1 : 1,000 |
| Mouse monoclonal anti-GST (Thermo Scientific, 740007M) | 1 : 5,000 |
| **Secondary antibodies (supplier)** | |
| Sheep anti-mouse IgG/HRP conjugated (GE Healthcare) | 1 : 10,000 |
| Donkey anti-rabbit IgG/HRP conjugated (GE Healthcare) | 1 : 10,000 |
