# Supplementary material for: Mammalian fatty acid synthase and O-GlcNAc transferase preferentially interact via their respective N-terminal regions
Source: Biochem Biophys Rep. 2026 Jan 6;45:102427. doi: 10.1016/j.bbrep.2025.102427 (PMC12808501; doi:10.1016/j.bbrep.2025.102427)
Supplement: Multimedia component 2 [file mmc2.docx]

**Supplementary table 2: Primers used in this study.**

| **Primer type** | **Name** | **Sequence (5’ → 3’)** |
| --- | --- | --- |
| **Cloning** | Hind III_*FASN*_Fw | CCCAAGCTTATGGAGGAGGTGGTGATTGCCGGC |
|  | *FASN*_Xba I_Rv | GCTCTAGATTACGTAGAATCGAGTCCGAGGAGAGGGTTAGGGATAG |
| **Mutagenesis** | *FASN* G826stop_Fw | AGTTCCCAGCTCCCCGATGAACTCCCCTCATCTC |
|  | *FASN* G826stop_Rv | GAGATGAGGGGAGTTCATCGGGGAGCTGGGAACT |
|  | *FASN* E973stop_Fw | CTCTTCGACCACCCGTAAAGCCCCACCCCCAACCCC |
|  | *FASN* E973stop_Rv | GGGGTTGGGGGTGGGGCTTTACGGGTGGTCGAAGAG |
|  | *FASN* W1634stop_Fw | CTGTCACCGGACTTCCTCTGAGATGTGCCTTCCAACTGG |
|  | *FASN* W1634stop_Rv | CCAGTTGGAAGGCACATCTCAGAGGAAGTCCGGTGACAG |
|  | *FASN* E1862stop_Fw | TTGCGGAGGAGCCGTAGGCAGTGTTGAAGGGGGCC |
|  | *FASN* E1862stop_Rv | GGCCCCCTTCAACACTGCCTACGGCTCCTCCGCAA |
|  | *FASN* C2202stop_Fw | GAGCTGGCATGACCCACGCCCAAGGAG |
|  | *FASN* C2202stop_Rv | CTCCTTGGGCGTGGGTCATGCCAGCTC |
| **Sequencing** | *FASN*_Fw1 | CGCCACCATCCTGAACG |
|  | *FASN*_Fw2 | TGATGACATCGTCCATTC |
|  | *FASN*_Fw3 | CTTCCCCAACGGTTCAG |
|  | *FASN*_Fw4 | GCTGAAGATGGTGGTGC |
|  | *FASN*_Fw5 | CTTGGTGAACTGTCTCC |
|  | *FASN*_Fw6 | GTCGCTTCCTGGAAATTG |
|  | *FASN*_Fw7 | GTGGAGACGATGAGCAC |
|  | hGH_PA_Rv | CAGCTTGGTTCCCAATAG |
