# Supplementary material for: Mammalian fatty acid synthase and O-GlcNAc transferase preferentially interact via their respective N-terminal regions
Source: Biochem Biophys Rep. 2026 Jan 6;45:102427. doi: 10.1016/j.bbrep.2025.102427 (PMC12808501; doi:10.1016/j.bbrep.2025.102427)
Supplement: Multimedia component 3 [file mmc3.docx]

**Supplementary table 3: Interface residues within 5A between OGT and FASN**

OGT

Chain A, Residue PRO 120

Chain A, Residue TYR 126

Chain A, Residue SER 148

Chain A, Residue GLN 151

Chain A, Residue TYR 152

Chain A, Residue GLU 174

Chain A, Residue GLU 175

Chain A, Residue LYS 177

Chain A, Residue ALA 178

Chain A, Residue LEU 181

Chain A, Residue LYS 182

Chain A, Residue ILE 184

Chain A, Residue GLU 185

Chain A, Residue TRP 194

Chain A, Residue LEU 197

Chain A, Residue PHE 201

Chain A, Residue GLU 206

Chain A, Residue TRP 208

Chain A, Residue LEU 209

Chain A, Residue HIS 212

Chain A, Residue HIS 213

Chain A, Residue LYS 216

Chain A, Residue ALA 238

Chain A, Residue ILE 240

Chain A, Residue ASP 242

FASN

Chain B, Residue HIS 350

Chain B, Residue GLY 351

Chain B, Residue LEU 352

Chain B, Residue GLN 379

Chain B, Residue PRO 380

Chain B, Residue LEU 381

Chain B, Residue PRO 382

Chain B, Residue VAL 383

Chain B, Residue ARG 384

Chain B, Residue PRO 410

Chain B, Residue PRO 411

Chain B, Residue PRO 412

Chain B, Residue ALA 413

Chain B, Residue PRO 414

Chain B, Residue ALA 415

Chain B, Residue PRO 416

Chain B, Residue HIS 417

Chain B, Residue ALA 418

Chain B, Residue THR 419

Chain B, Residue LEU 420

Chain B, Residue PRO 421

Chain B, Residue ARG 443

Chain B, Residue HIS 444

Chain B, Residue SER 445

Chain B, Residue GLN 446

Chain B, Residue ASP 447

Chain B, Residue GLY 474

Chain B, Residue LEU 508

Chain B, Residue MET 511

Chain B, Residue ARG 512

Chain B, Residue ASP 514

Chain B, Residue ARG 517

Chain B, Residue LEU 539

Chain B, Residue THR 541

Chain B, Residue ARG 825

Chain B, Residue GLY 826
